# Supplementary material for: Targeting Glioblastoma-Associated Macrophages for Photodynamic Therapy Using AGuIX®-Design Nanoparticles
Source: Pharmaceutics. 2023 Mar 20;15(3):997. doi: 10.3390/pharmaceutics15030997 (PMC10057379; doi:10.3390/pharmaceutics15030997)
Supplement: Supplementary file 1 [file pharmaceutics-15-00997-s001.zip › pharmaceutics-2249805-supplementary.pdf]

Supplementary Data :

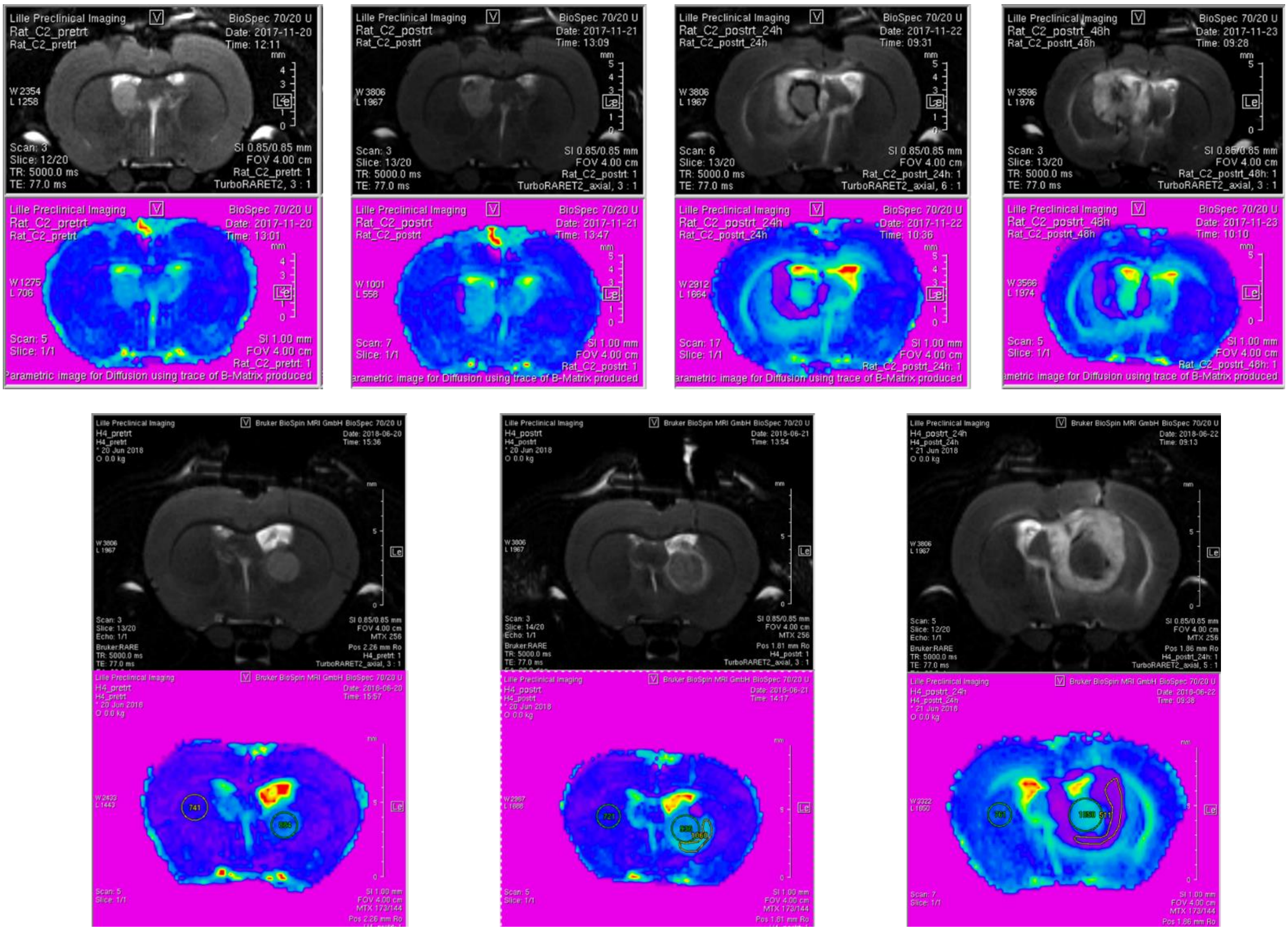

Figure S1. Two other rats treated by PDT after intravenous injection of AGuIX@PS@KDKPPR nanoparticles (1.75 µmol.kg^{-1} porphyrin equivalent) and followed by MRI analysis *via* T2 anatomical and diffusion (DWI - Diffusion Weighted Imaging) sequences.
